# Clinical Significance of Nontuberculous Mycobacteria Isolated From Respiratory Specimens in a Chinese Tuberculosis Tertiary Care Center

**DOI:** 10.1038/srep36299

**Published:** 2016-11-03

**Authors:** Hongfei Duan, Xiqin Han, Qingfeng Wang, Jing Wang, Jun Wang, Naihui Chu, Hairong Huang

**Affiliations:** 1 Department of Tuberculosis, Beijing Key Laboratory for Drug Resistant Tuberculosis Research, Beijing Chest Hospital, Capital Medical University, Beijing Tuberculosis and Thoracic Tumor Institute, Beijing, China; 2 National Clinical Laboratory on Tuberculosis, Beijing Key Laboratory for Drug Resistant Tuberculosis Research, Beijing Chest Hospital, Capital Medical University, Beijing Tuberculosis and Thoracic Tumor Institute, Beijing, China

## Abstract

The clinical relevance of non-tuberculous mycobacteria (NTM) has been reported to be different dramatically by species or by regions, however, no such evaluation has been performed in China.A retrospective study was performed in Beijing Chest Hospital. All the NTM strains isolated from respiratory specimens in the past 5 years, and patients’ clinical records (symptoms and radiographic information etc.) were investigated. The clinical relevance was evaluated according to the criteria recommended by the American Thoracic society. Totally 232 NTM strains were recruited, among them, *M. intracellulare* was the dominant species (40.5%), followed by *M. abscessus* (28.4%). 109 patients, with 185 total isolates, had full clinical records available for review. 84.4% (38/45), 85.7% (24/28%) and 63.6% (7/11) of patients with isolation of *M. intracellulare, M. abscessus* and *M. kansasii,* respectively, were categorized as definite NTM disease. Whereas all the 10 patients with isolation of *M. gordonae* were defined as unlikely NTM disease. The majority of NTMs isolates yielded from respiratory specimens in Beijing Chest Hospital were clinically significant, and *M. intracellulare* and *M. abscessus* was the dominated species of NTM lung disease. NTM lung infections demonstrated some specific chest radiograph characteristics.

In recent years, the frequency of nontuberculous mycobacteria (NTM) isolation from respiratory specimens, as well as the number of NTM infection patient, has increased rapidly in many countries^1^,^2^,^3^,^4^,^5^. Moreover, marked geographic variability was observed regarding both the prevalence of NTM lung disease and the mycobacterial species responsible for it^6^. Patients with NTM disease are often indistinguishable clinically or radiographically from patients infected with *Mycobacterium tuberculosis*^7^,^8^,^9^,^10^. However, the strategy of treatment and the prognosis of NTM disease can be significantly different from TB, resulting in different implications for public health.

In China, tuberculosis remains a serious public health problem and as a consequence, patients with positive outcomes of acid-fast bacilli (AFB) smear test or with isolation of mycobacteria by culture for sputum specimens have been routinely diagnosed as pulmonary tuberculosis and been administrated with anti-tuberculosis drugs^11^. The extent of misdiagnosis and inappropriate treatment of disease due to NTM infection in China lacks comprehensive understanding. On the other hand, NTM isolation does not affirmatively mean disease, so that the health providers have to stay alert for possible contamination or NTM colonization all the time. Thus the isolation of NTM species may pose a diagnostic difficulty for clinicians.

We performed an institution-based observational study for NTM lung disease in Beijing Chest Hospital (Beijing, China), and investigated the clinical, microbiological and radiographic information of possible NTM infection patients from 2010 to 2015. The purpose of the current study was to determine the isolation frequency of different NTM species from respiratory specimen over a 5-year period, and to evaluate their clinical significance.

## Materials and Methods

### Study subjects

All NTM strains isolated from respiratory specimens in Beijing Chest Hospital over a 5-year period from May 2010 to May 2015 were retrospectively analyzed. The medical records of patients from whom NTM strains isolated were retrospectively reviewed. Informed consent was obtained from all subjects included in the study. The protocols and procedures for the protection of human subjects were approved by the Ethics Committee of Beijing Chest Hospital. Furthermore, all the methods were carried out in accordance with the approved guidelines.

### Mycobacterium cultures

Sputum and bronchial washing specimens were decontaminated using the N-acetyl-L-cysteine 2% NaOH method. Löwenstein-Jensen medium culture or liquid culture using MGIT 960 system (BD Biosciences, Sparks, MD, USA), or both methods were performed to recover mycobacteria. Mycobacterial growth in PNB-containing medium was used as a presumptive test for NTM screening.

### Identification of NTM species

NTM isolates were identified to the species level by sequence alignment of 16S rDNA, 16-23S rRNA gene internal transcribed spacer (ITS), *rpoB* and *hsp65* genes. Preparation of the PCR amplification and DNA sequencing were done as described previously^12^.

### Clinical significance evaluation of isolated NTM

Patient with clinical record containing symptom, radiographic and microbiological information was considered clinically evaluable and classified as having definite, probable, or unlikely NTM lung disease according to the diagnostic criteria for NTM lung disease issued by American Thoracic Society(ATS) in 2007^13^. Definite NTM disease was defined as (1) Pulmonary symptoms, nodular or cavitary opacities on chest radiograph, or a high-resolution computed tomography scan that showed multifocal bronchiectasis with multiple small nodules; (2) Positive culture results from at least two separate expectorated sputum samples; Positive culture result from at least one bronchial wash or lavage; or transbronchial or other lung biopsy with mycobacterial histopathologic features (granulomatous inflammation or AFB) and positive culture for NTM or biopsy showing mycobacterial histopathologic features (granulomatous inflammation or AFB) and one or more sputum or bronchial washings that are culture positive for NTM; (3) Exclusion of tuberculosis and other illnesses that may produce similar symptoms and signs. Probable NTM disease was diagnosed if the patient met the third criterion and either of the other two criteria for definite NTM disease. NTM disease was judged to be unlikely if the patient did not meet any of the criteria for definite or probable NTM disease. In addition, *M. gordonae* and *M. terrae* isolations, well-known environmental contaminants were considered unlikely in relation to the lung diseases regardless of the other manifestations^13^. Definite and probable NTM lung diseases were regarded as clinically significant NTM lung infection^14^,^15^.

### Radiographic feature analysis of the patients with definite NTM lung disease

Two TB specialists simultaneously analyzed the radiographic features of chest computed tomography (CT) scan. The definite NTM lung disease was classified into three forms, according to CT findings. The upper lobe cavitary form was defined as a combination of cavities, consolidation, and pleural thickening in the upper lobes, regardless of reticulonodular opacities. The nodular bronchiectatic form was defined as unilateral or bilateral bronchiectasis and nodular changes without visible cavities in the upper lobes. For nodular bronchiectatic form, the implication of lingular segment or the right middle lobe was investigated. Patient’s disease remained unclassified if it belonged neither to the upper lobe cavitary nor the nodular bronchiectatic form. In the unclassifiable form, multifocal lobular or segmental consolidations usually comprised the predominant finding^13^.

### Statistical analysis

All statistical analyses were performed using SPSS22.0 software (SPSS Inc., Chicago, IL, USA). Comparisons of categorical variables and numerical variables were performed using the Pearson Chi-squared tests or *t*-tests respectively. *P* values of less than 0.05 were considered to indicate statistical significance.

## Results

### Frequency of isolated NTM species

From May 2010 to May 2015, a total of 232 NTM isolates were reported from respiratory specimens which included *M. intracellulare* (n = 94, 40.5%), *M. abscessus* (n = 66, 28.4%), *M. kansasii* (n = 23, 9.9%), *M. fortuitum* (n = 20, 8.6%), *M. avium* (n = 11, 4.7%), *M. gordonae* (n = 10, 4.3%), *M. szulgai* (n = 3, 1.3%), *M. terrae*(n = 2, 0.9%), *M. simiae* (n = 1, 0.4%), *M. parascrofulaceum* (n = 1, 0.4%) and *M. neoaurum* (n = 1, 0.4%). 226 strains were isolated from sputa, 5 strains from bronchial washings and 1 strain from lung tissue.

### Clinical significance of NTM isolates

Among those 232 isolates, 109 patients which accounted for 185 NTM isolates had full clinical records available for review. All patients were free of human immunodeficiency virus infection. Patients were then grouped into the three disease categories based on criteria cited above. Of the 109 patients, 72 (66.1%) had definite diseases, 27 (24.8%) had probable disease, and 10 (9.2%) had unlikely disease. *M. gordonae* was isolated from sputum sample of one patient who acquired *M. abscessus*-associated lung disease during antibiotic treatment. This strain was excluded from further analysis. The implicated NTM organisms are shown in Table 1.

NTM isolation demonstrated extremely high clinical significance among the 109 enrolled patients as 99 (90.8%) were defined as definite or probable disease. The absolute majority of patients with isolation of *M. intracellulare* (84.4%), and *M. abscessus* (85.7%) were finally categorized as definite disease, while all the isolations of *M. intracellulare*, *M. abscessus, M. kansasi, M. kansasii, M. avium* and *M. szulgai* were defined as clinically significant. On the other hand, none of the 10 patients, with *M. gordonae* isolation, were considered to have relation with disease.

### Clinical and radiographic characteristics of patients with definite lung disease

Characteristics of the patients in current study are summarized in Table 2. There were no significant differences in the sex, mean age, body mass index (BMI), presence of underlying disease and symptom among the three groups. Sputum AFB smears were positive for 84.7% (61/72) of the patients with definite NTM lung disease, 67.9% (19/28) with probable disease, whereas only 1 out of the 10 patients with unlikely disease produced positive smear test outcome.

**Figure 1:**
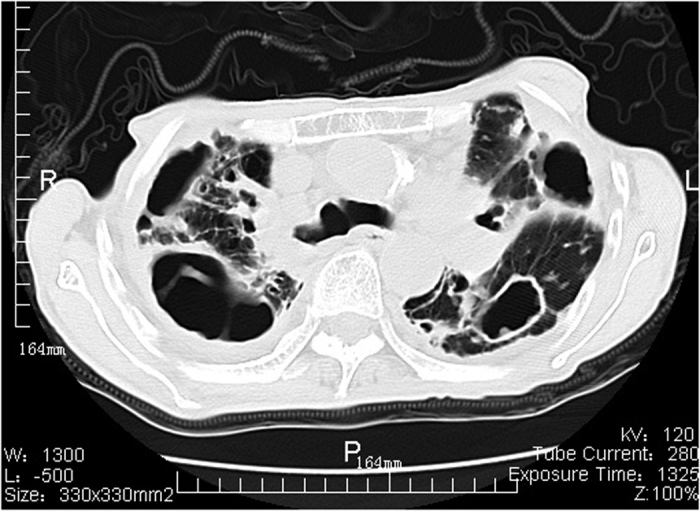
*M. intracellulare* lung disease in a 68-year-old man. Chest CT scan shows large multi-cavitary lesions in bilateral upper lobes and pleural thickness.

**Figure 2:**
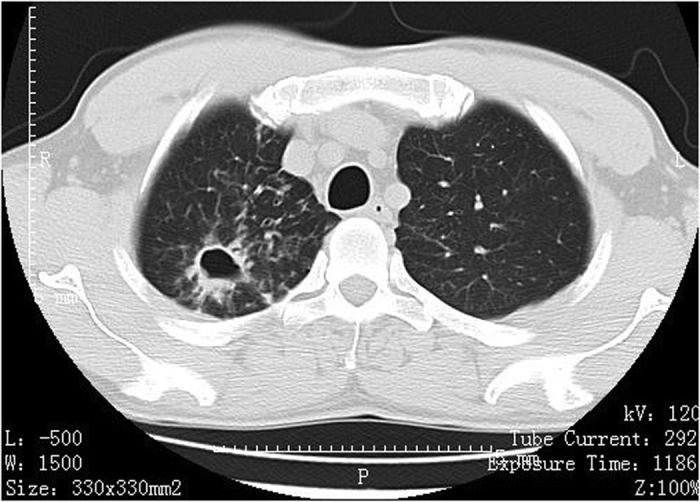
*M. kansasii* lung disease in a 46-year-old man. Chest CT scan shows a cavity and centrilobular nodules in right upper lobe.

**Figure 3:**
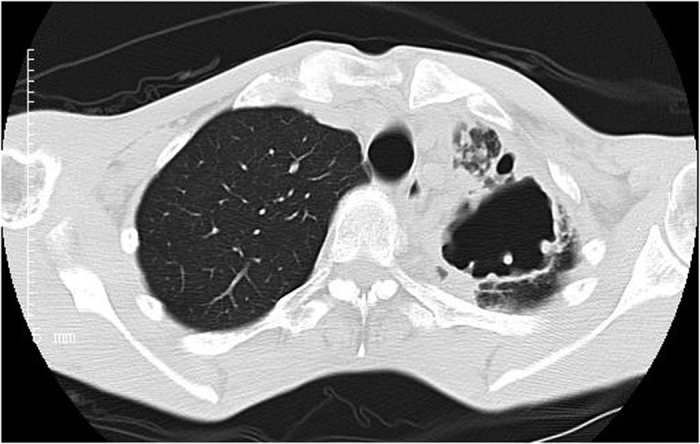
*M. szulgai* lung disease in a 38-year-old man. Chest CT scan shows a large cavity and centrilobular nodules in left upper lobe and pleural thickness.

**Figure 4:**
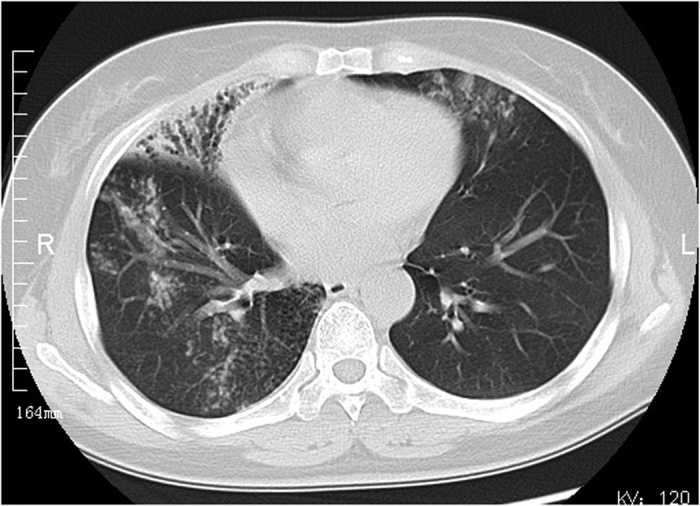
*M. intracellulare* lung disease in a 57-year-old woman. Chest CT scan shows centrilobular nodules and bronchiectasis. Also note lesions predominate in lingular segment and right middle lobe.

**Figure 5:**
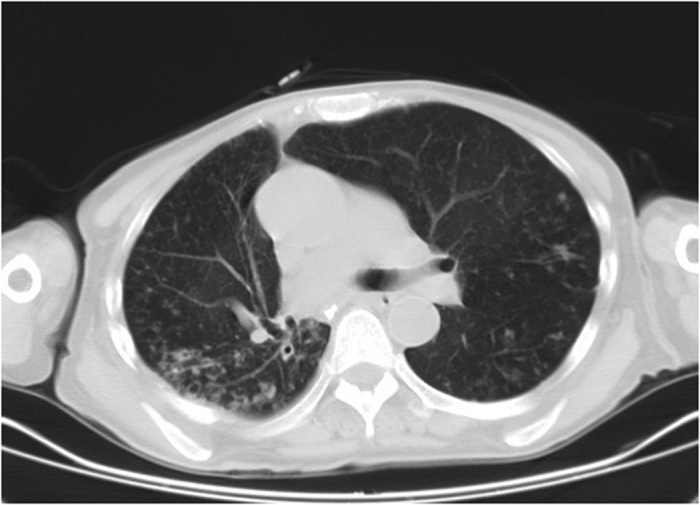
*M. abscessus* lung disease in a 62-year-old woman. Chest CT scan shows centrilobular nodules and bronchiectasis. Also note lesions without segment or lobe predominance.

Among 72 patients with definite NTM disease, 29 had the upper lobe cavitary form (Figs 1, 2, 3), 41 patients had nodular bronchiectatic form (Figs 4 and 5), whereas 2 patients possessed the unclassifiable form (Table 3). For 27 cases with probable NTM lung disease, 8 cases classified as unclassifiable form. Whereas among the 10 cases with unlikely NTM lung disease, 9 of had unclassifiable form. For nodular bronchiectatic form patients, *M. intracellulare* lung disease had more tendency to infect the lingular segment and the right middle lobe compared with *M. abscessus* lung disease (72.7% (16/22) vs 26.3% (5/19), p < 0.05).

## Discussion

NTM have been implicated in an increasingly large proportion of pulmonary disease throughout the world, in both immunocompetent and immunocompromised hosts^16^,^17^,^18^,^19^. In many countries, MAC (including *M. intracellulare* and *M. avium*) are the most common NTM isolates. On the other hand, *M. avium* is predominant in North- and South America, *M. intracellulare* is most frequently isolated in Australia-Queensland, South Africa and some provinces in China^6^,^20^,^21^,^22^. Our study showed that *M. intracellulare* was the most common species of NTM isolation, followed by *M. abscessus* in Beijing Chest Hospital which is located in north of China.

It is believed that NTM disease is under-reported in the tuberculosis-endemic countries. The main reason could be the high burden of tuberculosis which attracts bulk of the attention of clinicians. In our knowledge, this is the first research to document the clinical significance of NTM isolates from respiratory specimens in China, a high TB burden country. The major findings of this study are as following: (1) NTM strains isolated from respiratory specimens demonstrated very good relation with lung infections (90.9%). (2) *M. intracellulare* was the most common NTM species (40.5%) isolated from clinical respiratory specimens, and it was also the most common pathogen (52.8%) of NTM lung disease in our institution. Although in some areas *M. intracellulare* is less likely to cause NTM lung disease^23^, our data demonstrated that *M. intracellulare* is the predominant bacteria for NTM lung disease in Beijing Chest Hospital. (3) *M. abscessus* was the second most commonly isolated species (28.4%) and represented the second most common pathogen (33.3%). Since *M. intracellulare* and *M. abscessus* lung diseases had the most poor prognosis among all the NTM lung diseases, according to our outcomes, the NTM lung disease treatment will be very challenging in China as those two species account for the majority of NTM lung diseases^13^,^24^.

Although the incidence of pulmonary disease caused by *M. kansasii* is the second most common (approximately 20%) type of NTM pulmonary disease in Japan and USA^25^,^26^, it is uncommon in our institution. However, in Shanghai, another industrialized city in the south of China, *M. kansasii* is the most frequently identified isolate, accounting for 45% of all NTM isolates^20^. It seems that geographic distribution of NTM species is significantly different in a large country like China.

Among the definite, probable and unlikely NTM lung disease, there were no significant differences in the sex, mean age, BMI, presence of underlying disease and symptoms, but fewer patients with unlikely NTM lung disease were sputum AFB smear positive. It is possible that unlikely NTM lung diseases have less bacterial load, however with a more sensitive method, such as liquid culture, the chances to recover it can increase. 8 out of the 10 unlikely NTM infection cases had isolations recovered by MGIT960 only, which was consistent with this assumption. At a hospital in USA, the dramatic increase of NTM isolation was attributed to the more sensitive liquid culture technique, which led to less clinical significance of NTM isolations^27^. All the 10 unlikely NTM lung disease cases had the isolations of *M. gordonae* in this study, which warns clinicians to interpret NTM recovery by MGIT960 systems with cautions, especially for *M. gordonae*.

Compared with reports from other countries^14^,^15^,^23^, nearly 66.1% of the patients with NTM isolations were diagnosed as definite NTM disease in this assay, which is much higher than those of most other studies. Furthermore, we speculated that some more patients with possible NTM lung disease due to less bacterial evidence would fulfill the definite NTM lung disease criteria during the follow-up process. We attributed the tight relationship of NTM isolation with lung infection mainly to more severe cases enrolled in the study. Our institution is the top tuberculosis referral center in China and has admitted various patients with serious symptoms transferred from other hospitals. In addition, another contributing factor may be that clinicians in our hospital were very knowledgeable of NTM disease, hence they regularly prescribed repeated mycobacterium culture for suspected NTM disease patients. The only species we did not find good clinical significance was *M. fortuitum*. A study in Japan also found that among 26 patients with a minimum of two *M. fortuitum* isolations, none showed clinical aggravation during the follow-up period^28^. It is likely that colonization or contamination during culture happens frequently with this species.

NTM lung infection may have some specific image characteristics for a CT scan. Among patients with definite NTM lung disease, fewer cases have unclassifiable form for radiographic features compared with patients with probable and unlikely NTM disease. Based on our research, cases without typical nodular bronchiectatic and upper lobe cavitary forms are less likely to be related to NTM lung disease. The position of nodular bronchiectatic lesion demonstrated species-specific characteristics. *M. intracellulare* lung diseases were more likely to be implicated in the lingular segment and the right middle lobe in contrast with *M. abscessus* lung diseases.

## Conclusions

In conclusion, a substantial proportion (90.9%) of patients, from whom NTM isolates were recovered, exhibited definite or probable NTM lung disease in our institution. The most common etiologies of NTM lung disease included *M. intracellulare* and *M. abscessus*. NTM lung infections frequently demonstrated the upper lobe cavitary form or nodular bronchiectatic form for chest radiograph examination. However, as NTM only accounted for 2.6% of all the mycobacterial isolations in our hospital according to previous work^29^, the clinical significance in other areas of China with high percentages of NTM isolation and different species constitutions might be different with our conclusions, but more investigations need be done to prove this assumption.

## Additional Information

**How to cite this article**: Duan, H. *et al*. Clinical Significance of Nontuberculous Mycobacteria Isolated From Respiratory Specimens in a Chinese Tuberculosis Tertiary Care Center. *Sci. Rep.* **6**, 36299; doi: 10.1038/srep36299 (2016).

**Publisher’s note:** Springer Nature remains neutral with regard to jurisdictional claims in published maps and institutional affiliations.

## Figures and Tables

**Table 1:** Etiology of Clinically Significant NTM Lung Infection based on per-patient analysis.

Organism	Classification of NTM lung Disease			
	Definite NTM Lung Disease, No. (%)	Probable NTM Lung Disease, No. (%)	Unlikely NTM Lung Disease, No. (%)	Total, No.
*M. intracellulare*	38 (84.4%)	7 (15.6%)	0	45
*M. abscessus*	24 (85.7%)	4 (14.3%)	0	28
*M. kansasii*	7 (63.6%)	4 (36.4%)	0	11
*M. fortuitum*	0	8 (100%)	0	8
*M. avium*	2 (33.3%)	4 (66.7%)	0	6
*M. szulgai*	1 (100%)	0	0	1

**Table 2:** Characteristics of 109 patients from whom NTM were isolated.

Characteristics	Classification of NTM lung Disease		
	Definite NTM Lung Disease (n = 72)	Probable NTM Lung Disease(n = 27)	Unlikely NTM Lung Disease (n = 10)
Male	38	14	5
Mean age (y) ± SD	54.1 ± 22.5	51.8 ± 24.0	51.5 ± 24.1
BMI, kg/m^2^	22.3 ± 2.7	24.0 ± 2.6	23.9 ± 2.8
Underlying Disease			
Previous tuberculosis	11	3	0
COPD	6	4	3
Bronchiectasis	5	2	1
Silicosis	2	0	0
Diabetes Mellitus	4	2	1
Other disease	2	0	0
Previous steroid treatment	2	0	0
Smoker	7	4	4
Positive sputum AFB smear*	61	19	1
Symptom			
Cough	72	24	9
Productive cough	35	13	4
Hemoptysis	25	9	1
Constitutional symptom	4	1	0
Type of disease			
Nodular bronchiectatic form	41	14	1
Upper lobe cavitary form	29	5	0
Unclassifiable form*	2	8	9

^*^P < 0.05.

**Table 3:** Characteristics of 72 patients with definite NTM lung disease according to species.

Characteristics	Etiology of NTM lung Disease				
	*M. intracellulare* (n = 38)	*M. abscessus* (n = 24)	*M.kansasii* (n = 7)	*M.avium* (n = 2)	*M.szulgai* (n = 1)
Male	21	9	5	1	1
Mean age (y) ± SD	52.1 ± 16.5	46.8 ± 17.1	53.5 ± 10.9	38.6	41
BMI, kg/m^2^	21.2 ± 1.6	23.0 ± 2.1	22.7 ± 2.7	22.5	20.1
Underlying Disease					
Previous tuberculosis	6	4	1	0	0
COPD	3	2	0	1	0
Bronchiectasis	2	2	0	1	0
Silicosis	2	0	0	0	0
Diabetes Mellitus	2	2	0	0	0
Other disease	2	0	0	0	0
Previous steroid treatment	2	0	0	0	0
Previous smoking	6	1	0	0	0
Positive sputum AFB smear	32	21	6	1	1
Symptom					
Cough	38	24	7	2	1
Productive cough	15	13	5	1	1
Hemoptysis	15	5	3	2	0
Constitutional symptom	3	1	0	0	0
Type of disease					
Nodular bronchiectatic form	22	19	0	0	0
Upper lobe cavitary form	16	3	7	2	1
Unclassifiable form	0	2	0	0	0
